# Transformation of human bronchial epithelial cells alters responsiveness to inflammatory cytokines

**DOI:** 10.1186/1471-2407-5-145

**Published:** 2005-11-04

**Authors:** Gregory M Loewen, Erin Tracy, Frédéric Blanchard, Dongfeng Tan, Jihnhee Yu, Sameera Raza, Sei-Ichi Matsui, Heinz Baumann

**Affiliations:** 1 Departments of Medicine, Roswell Park Cancer Institute, Buffalo, NY 14263, USA; 2 Departments of Molecular and Cell Biology, Roswell Park Cancer Institute, Buffalo, NY 14263, USA; 3 Departments of Pathology, Roswell Park Cancer Institute, Buffalo, NY 14263, USA; 4 Departments of Biostatistics, Roswell Park Cancer Institute, Buffalo, NY 14263, USA; 5 Departments of Cancer Genetics, Roswell Park Cancer Institute, Buffalo, NY 14263, USA

## Abstract

**Background:**

Inflammation is commonly associated with lung tumors. Since inflammatory mediators, including members of the interleukin-6 (IL-6) cytokine family, suppress proliferation of normal epithelial cells, we hypothesized that epithelial cells must develop mechanisms to evade this inhibition during the tumorigenesis. This study compared the cytokine responses of normal epithelial cells to that of premalignant cells.

**Methods:**

Short-term primary cultures of epithelial cells were established from bronchial brushings. Paired sets of brushings were obtained from areas of normal bronchial epithelium and from areas of metaplastic or dysplastic epithelium, or areas of frank endobronchial carcinoma. In 43 paired cultures, the signalling through the signal transducer and activator of transcription (STAT) and extracellular regulated kinase (ERK) pathways and growth regulation by IL-6, leukemia inhibitory factor (LIF), oncostatin M (OSM), interferon-γ (IFNγ) or epidermal growth factor (EGF) were determined. Inducible expression and function of the leukemia inhibitory factor receptor was assessed by treatment with the histone deacetylase inhibitor depsipeptide.

**Results:**

Normal epithelial cells respond strongly to OSM, IFNγ and EGF, and respond moderately to IL-6, and do not exhibit a detectable response to LIF. In preneoplastic cells, the aberrant signaling that was detected most frequently was an elevated activation of ERK, a reduced or increased IL-6 and EGF response, and an increased LIF response. Some of these changes in preneoplastic cell signaling approach those observed in established lung cancer cell lines. Epigenetic control of LIF receptor expression by histone acetylation can account for the gain of LIF responsiveness. OSM and macrophage-derived cytokines suppressed proliferation of normal epithelial cells, but reduced inhibition or even stimulated proliferation was noted for preneoplastic cells. These alterations likely contribute to the supporting effects that inflammation has on lung tumor progression.

**Conclusion:**

This study indicates that during the earliest stage of premalignant transformation, a modified response to cytokines and EGF is evident. Some of the altered cytokine responses in primary premalignant cells are comparable to those seen in established lung cancer cell lines.

## Background

Lung epithelial cells are consistently exposed to many irritants and pathogens. Excessive exposure can lead to inflammatory conditions even though effective mechanisms are in place to contain and remove harmful components [1]. Epithelial damage results in tissue repair. Chronic injury and repeated cycles of tissue repair in presence of an inflammatory reaction may provide conditions that are conducive for selection of cells with enhanced proliferation and/or reduced sensitivity to signals for growth arrest and differentiation [2]. An environment that favors tumorigenesis is created when genetic and epigenetic changes enhance proliferation, reduce differentiation and/or attenuate apoptotic reactions [3]. Changes in epithelial morphology and proliferation may result in decreased autofluorescence that is grossly detectable with autofluorescence bronchoscopy [4]. A step-wise progression has been hypothesized to precede frank malignancy [5,6], and recent autofluorescence bronchoscopy studies have confirmed the malignant potential of metaplasia and dysplasia of the bronchial epithelium [7-9]. Direct visualization of these changes has made it possible to better understand the role of inflammation in lung carcinogenesis.

Inflammation has been reported to contribute to the development of cancer [1,2,10,11], and IL-6 cytokines, such as oncostatin M (OSM), actually arrest growth of cultured epithelial [12] and other cell types [13]. We hypothesized that members of the interleukin-6 (IL-6) cytokine family may contribute to the step-wise progression by providing growth-stimulatory activity. We also hypothesized that the transformed premalignant cells escape the inhibitory activity of cytokines as a function of the transformation process and that these transformed cells have altered cytokine responsiveness. These alterations should include reduced signaling through growth-suppressing pathways and/or enhanced signaling through growth-promoting pathways.

IL-6 cytokines are recognized by receptors that belong to the group of hematopoietin receptors [14]. Signal transduction is communicated by receptor-associated Janus protein tyrosine kinases that phosphorylate the receptor subunits. The signaling proteins are recruited to the tyrosine phosphorylated receptors, include signal transducer and activator of transcription-3 (STAT3), the protein tyrosine phosphatase SHP-2 and the adaptor Shc, which link to the RAS-MAPK-ERK pathway [14,15]. The magnitude of these immediate signaling reactions is a measure for the level of receptor activation in treated cells, and this is particularly true for the tyrosine phosphorylation of STAT3 and dual phosphorylation of ERK1/2 [16].

At the present time, very little is known about: (a) the response pattern of normal, non-immortalized human lung epithelial cells to inflammatory mediators, (b) the individual variation of the response patterns, and (c) the effects that premalignant transformation has on the responsiveness. Our study was designed to determine the response of bronchial epithelial cells from normal epithelium and abnormal lesions to inflammatory mediators and IL-6-type cytokines, and to define the effects of these cytokines on signaling and cell growth regulation. We developed techniques for obtaining proliferating epithelial cell cultures from bronchoscopy brushings and we used those cultures for functional screening.

## Methods

### Patient population

Patients with tobacco exposure who were enrolled in an institutional lung cancer screening trial underwent autofluorescence bronchoscopy using the LIFE system (LIFE systems, Xillix, Vancouver BC) in concert with low-dose spiral CT of the chest [17]. Informed consent was obtained and procedures were performed in accordance with an IRB-approved protocol.

### Bronchoscopy and cytologic brushing

Bronchoscopy was performed under monitored anesthesia care. The airways were examined with white light and for autofluorescence. The appearance of the bronchial mucosa was classified as normal or abnormal. Bronchial epithelial brushings (area of ~25 mm^2^) and biopsies were taken from one normal and one to three abnormal sites. Cytology samples from the brushings were directly smeared onto glass slides and fixed in 95% ethanol. Residual tissue on the cytology brush was then used to establish cell cultures. Diagnoses of metaplasia, grades of dysplasia or non-invasive carcinoma *in situ *were made strictly based on the criteria established by the World Health Organization [18].

### Primary epithelial cell cultures from bronchial brushing

The cytology brush with adherent tissue was incubated in 5 ml of trypsin (1/125 dilution in PBS; Invitrogen, Carlsbad, CA) at 37°C for 5 min. Dissociated cells were collected by centrifugation, resuspended in serum- and calcium-free keratinocyte medium containing human recombinant EGF, bovine pituitary extract, cholera toxin, and mycostatin (Invitrogen), and plated into one 3.5-cm diameter, collagen type-1-coated dish. The number of adherent cells after 24 h incubation ranged from <1 to ~20 × 10^3^. Medium was replaced every 4 days. When successful (generally with >5 × 10^3 ^cells initially present), proliferating epithelial cells reached 80% confluence (~1 × 10^6 ^cells) in 2–3 weeks. Subcultures of the first passage (in 24-well culture plate) were used to determine the cytokine response patterns and cellular homogeneity by immunostaining for expression of cytokeratin (class AE1/3 pancytokeratin). Cultures from the second passage were used for thymidine incorporation. Cells from the third passage were used for confirming consistency of cytokine response profile and spectral karyotyping (SKY) [19]. Of note is that all primary epithelial cultures showed limited life span and ceased to divide after 4 to 7 passages.

### Isolation of macrophages, fibroblasts, and type II epithelial cells

Human residual lung tissue was obtained from Roswell Park Cancer Institute Tissue Procurement Service under an IRB-approved protocol. Macrophages were mechanically extracted from minced lung tissue, purified by Histopaque gradient centrifugation (Sigma Chemical Co., St. Louis, MO) and plated at a density of 3 × 10^5 ^cells/cm^2 ^in RPMI-1640 containing 10% FCS. After 45 min, adherent cells (>95% macrophages) were incubated in medium (1 ml/1 × 10^6 ^cells) containing 1 μg/ml LPS. Conditioned medium was collected after 16 h. The concentrations of cytokines were determined by multiplex immunobead flow cytometry (Luminex Inc., Austin, TX). To obtain fibroblasts, macrophage-depleted lung tissue pieces were incubated with RPMI-1640 containing 10% FCS, antibiotics and mycostatin. After 7–10 days, fibroblasts had grown out of the pieces to ~50% confluence. These were selectively released by digestion with trypsin for 2–5 min at room temperature. Fibroblasts from passage 2 were used for analysis. To obtain type II epithelial cells, macrophage-depleted residual lung tissue pieces were incubated with 5 volumes of trypsin for 15 min at 37°C. Trypsin digestion was repeated. Cells released during the second digestion were cultured as described for bronchial epithelial cells. The homogeneity of epithelial cell population was determined by immunostaining of first passage cultures for surfactant expression (Dako, Captine, CA) [20].

### Established lung cell lines

Bronchial epithelial cell lines immortalized with the human papilloma virus E6E7 gene, HBE4 and HBE137 (ATCC, Manassas, VA), were cultured like primary bronchial epithelial cells. The non-small cell lung cancer cell line A549 was grown in DMEM containing 10% FCS, and the NCI lines H23, H125, H358, H441, H522 (ATCC) and ADLC [21] were cultured in RPMI-1640 medium containing 10% FCS.

### Cytokine treatments for analysis of signalling

Cells were seeded into 24-well plates. When the cultures reached ~90% confluence, they were incubated for 2 h in serum- and factor-free RPMI-1640 (to reduce signalling effects by serum growth factors or EGF), followed by treatment for 15 min with the same medium containing 100 ng/ml recombinant IL-6, OSM (Amgen Corporation, Seattle, WA), LIF (Wyeth Pharmaceuticals, Cambridge MA), EGF (Gibco-Invitrogen Carlsbad, CA), 100 units/ml IFNγ (Roche Applied Science, Indianapolis, IN), 500 ng/ml insulin or 0.1 μM phorbol myristic acid (Sigma, St. Louis, MO). Cells were washed with PBS and lysed with RIPA buffer containing 0.1 mM orthovanadate and 1:100 diluted protease inhibitor cocktail (Calbiochem, San Diego, CA). LIF receptor (LIFR) expression was induced by treating epithelial cells with growth medium containing 20 nM of the histone deacetylase inhibitor depsipeptide FR901228 (NCI) [22].

### Growth analysis

Cells were seeded at 5% confluence (~1–5 × 10^3 ^primary epithelial cells, HBE135 and fibroblasts) or 1% confluence (~0.5–4 × 10^3 ^cells from established lines) into 24-well culture plates using the appropriate complete growth medium. Replicate cultures were treated with complete growth medium containing serially diluted cytokines or conditioned medium of LPS-activated macrophages. At day 3, the media were replaced and at day 6, the cells were released by trypsin digestion and the number of viable (trypan blue dye-excluding) cells counted in a hemocytometer. In each series, the mean number of cells recovered for cultures treated with growth medium alone (ranged from 0.5 to 4 × 10^5^) was used as used as internal reference (defined as 100%).

### Thymidine incorporation

Epithelial cells were seeded with growth medium into 24-well culture plates (~5 × 10^4 ^cells per well). After 24 h, duplicate cultures were treated with growth medium containing serially diluted OSM or conditioned medium from LPS-activated macrophages. Twenty-four hours later, 1 μCi of [^3^H]thymidine (Amersham Biosciences) was added to each culture and incubation of the cells continued for an additional 16 h. Cells were released by trypsin and collected onto filter paper by a cell harvester (Tomtec, Hamden, CT). The amount of incorporated tritium was measured by a scintillation counter (Trilux microbeta, Turku Finland). The mean of the net values of the duplicate wells was expressed relative to the mean incorporation determined for the control cultures in each of the series, which was defined as 100%.

### Western blot analysis

Replicate aliquots of cell lysates, containing 10 μg of protein, were electrophoresed on 7.5% polyacrylamide gels [16]. Extracts from paired samples were co-analyzed. The proteins were transferred to protean membranes (Schleicher & Schuell, Keene, NH). Immediately after transfer, the membranes were stained with Ponceau red to verify equal loading and membrane-transfer of proteins. In each experimental series, two replicate blots were horizontally cut at the ~60 kD size position; the upper sections were used to probe for phosphorylated and total STAT3 and the lower sections for phosphorylated ERK and total ERK. Separate blots were used to probe for phosphorylated and total STAT1. In cases where larger higher amounts of cells were recovered, extracts were also analyzed for receptor proteins. The membranes were reacted with antibodies to phospho-specific forms or ERK1/2, STAT1, STAT3 and EGFR (Cell Signalling Technology, Inc., Beverly, MA) and total forms of ERK1/2, STAT1, and STAT3, LIFR (Santa Cruz, Santa Cruz, CA). The membranes were incubated with the appropriate peroxidase-conjugated secondary antibodies (ICN Biomedical, Aurora, OH) and the antibody binding was visualized by enhanced chemiluminescence reaction (Amersham Biosciences Piscataway, NJ). In each experimental series, immunoblots were exposed to X-ray films for various lengths of time (1 sec to 30 min) to obtain images that are in the linear range of signal detection [23]. For pictorial presentation of immunoblot data, the signals for either total STAT3 or ERK1/2 served as loading controls.

### Densitometric analysis

Immunoblots were digitalized and quantified with Image Quant TL Software (Amersham Biosciences Piscataway, NJ). The net pixel value for each protein band that lied within the linear range of detection was normalized to the co-analyzed standard. To compare the responses between normal and abnormal epithelial cell cultures from individual donors, as well the responses among different donors, the OSM-induced phosphorylation of STAT1, STAT3 and ERK1/2 in the normal cell cultures of each paired set was used as an internal reference (defined as 100). Similarly, the magnitude of increased or decreased signalling in cells from paired cultures was defined by the ratio with the values obtained for the cells derived from the bronchial sites initially identified as being normal. Since the level of phosphorylated STAT3 in LIF-treated normal cells was generally undetectable, we arbitrarily used 1% as lowest value for calculating fold changes. Thus, the calculated values for LIF effects may represent underestimates. Basal levels of signalling and cytokine responses of cell lines were determined by the level of phosphorylated STAT3 and ERK1/2 and normalized to that values obtained for the same cells treated with OSM (defined as 100%).

### Statistical evaluation

The inferences for all the hypothesis tests are based on the significance level of 0.05. All statistical analyses were performed in an exploratory manner. For each combination of biomarker (P-STAT1, P-STAT3, and P-ERK) and treatment (one control and 5 cytokines), the relative increase in phosphorylation in the abnormal cells compared with its matched normal cells from the same patient was subjected to the exact sign test with paired samples. A decrease or increase of DNA syntheses within a treatment (OSM or macrophage factors) by different doses of the treatment was tested using the exact sign test with paired samples and the exact Page's L test. The DNA synthesis values of normal and metaplastic cell cultures in paired samples were compared using the exact Wilcoxon two-sample test.

## Results

### Transformed lung epithelial cell lines have grossly abnormal patterns of cytokine responsiveness

**Figure 1:**
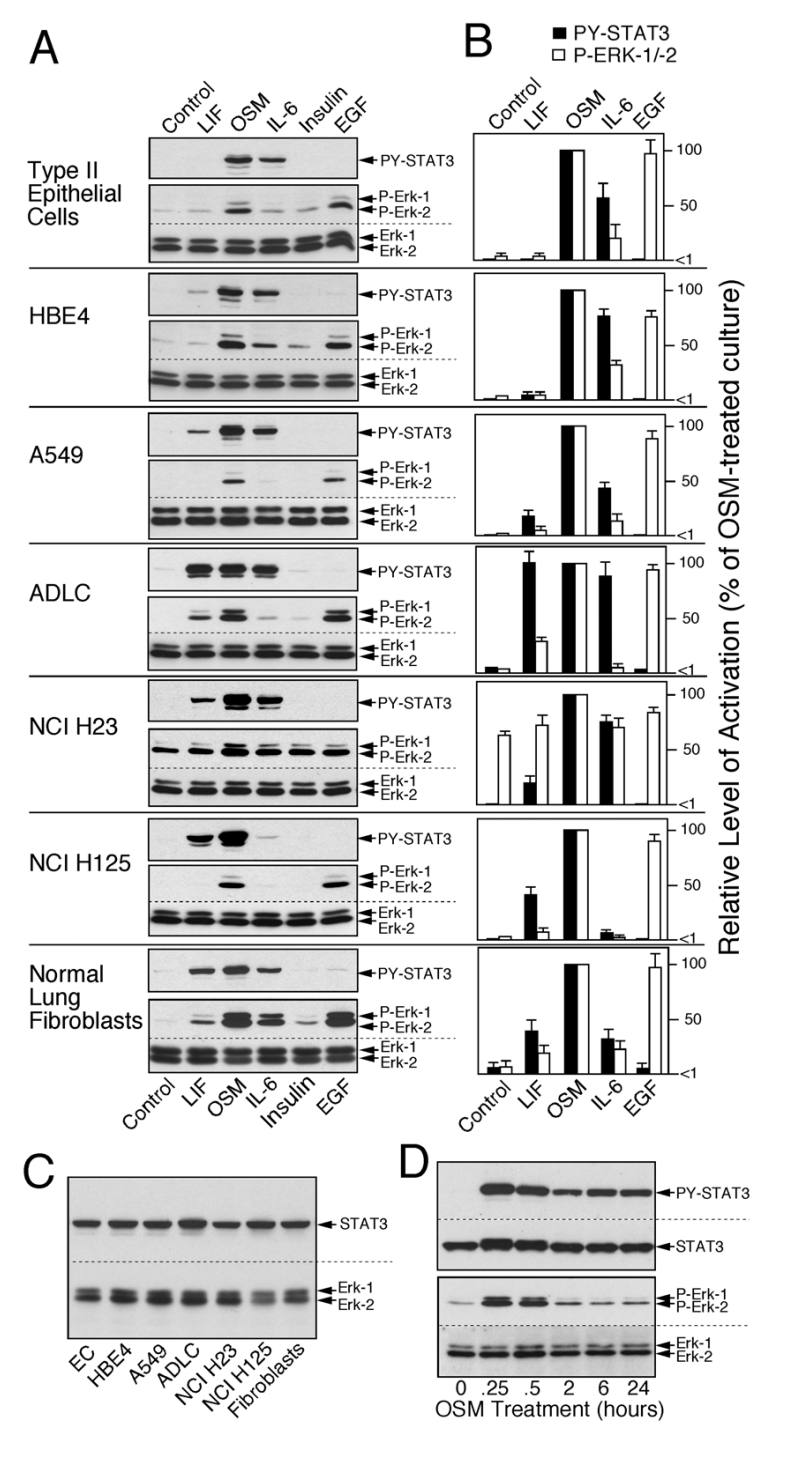
**Cytokine response of lung cells**. **A**. Primary cultures of the normal type II epithelial cells and fibroblasts from residual lung tissue and the indicated cell lines were treated for 15 min with factors listed at the bottom. Extracts were analyzed by immunoblotting for phosphorylated STAT3 and ERK-1/-2. The level of immunodetectable total ERK-1/-2 indicates protein loading. **B**. The quantitative values (mean and SD) for the phosphorylation of ERK-1/-2 (open bars) and STAT3 (closed bars) by treatments with LIF, OSM, IL-6 and EGF were determined in 5 independent experiments (expressed relative to the OSM effect, equal 100%). **C**. Extracts from control treated cultures from A were analyzed for the immunodetectable level of total STAT3 and ERK-1/-2. **D**. Type II epithelial cells were treated for the indicated length of time with OSM (100 ng/ml) and the level of phosphorylated STAT3 and ERK-1/-2 was determined by immunoblotting.

We predicted that if oncogenic transformation of lung epithelial cells is associated with altered responsiveness to inflammation and reduced growth-inhibition, then the most prominent deviations from the regulatory phenotype should be seen in advanced lung cancer cells. Therefore, as first step, we determined if responsiveness to members of the IL-6 family (IL-6, OSM and LIF) were detectable in *established *lines of malignant human non-small cell lung carcinoma cells. The responsiveness to the cytokines was defined by measurable phosphorylation of STAT and ERK. This is a treatment reaction that is a measure for the level of active cytokine receptors and downstream signaling pathways in the target cells [14,16]. Normal type II epithelial cells and pulmonary fibroblasts were included in the analyses to gauge cell type-specific differences in signaling reactions (Fig. 1).

Phosphorylation of STAT3 and ERK was sufficient for identifying responsiveness to cytokines in specific cell types (Fig. 1A shows representative immunoblots and Fig. 1B presents the quantitative data from independently performed experiments involving 5 separate matched cultures of type II epithelial cells and fibroblasts). In brief, normal type II epithelial cells showed the following features (Fig. 1A, top panel): OSM strongly activated phosphorylation of STAT3 and ERK, while IL-6 was less effective, and LIF did not produce any detectable response. EGF stimulated phosphorylation of ERK in the range of OSM, while insulin was minimally effective. Fibroblasts exhibited a prominent LIF response and a several-fold higher activation of ERK relative to STAT3 by IL-6 cytokines (Fig. 1A, bottom panel), in contrast to normal epithelial cells. The maximal level of receptor action in epithelial cells was reached after 15 min agonist treatment (Fig. 1D, example of OSM on normal type II epithelial cells).

Deviations from the normal regulatory phenotype of epithelial cells were already evident in immortalized bronchial epithelial cells, HBE4 (Fig. 1A) and HBE137 (not shown). These cell lines responded to IL-6 cytokines like normal epithelial cells, but showed a detectable STAT3 signaling by LIF and a ~2-fold higher ERK activation by OSM and IL-6. More profound differences were detected in carcinoma lines (Fig. 1A and not shown). In carcinoma cell lines, the major trends included a strong STAT3 activation by LIF (ADLC, H125), an increased (ADLC, H23) or decreased STAT3 activation by IL-6 (H125, H324), a decreased ERK activation by EGF (H522), and a treatment-independent, constitutive activation of the ERK pathway (H23). Despite the substantial changes in response patterns, STAT and ERK signaling in response to OSM was consistently high in all analyzed lung cancer cell lines. Moreover, the cell-type and line specific differences in STAT3 and ERK signaling were not due different expression of the signaling proteins as demonstrated by the comparable expression level of total STAT3 and ERK proteins among the cell types (Fig. 1C).

### Cell line-specific effect of cytokines on proliferation

**Figure 2:**
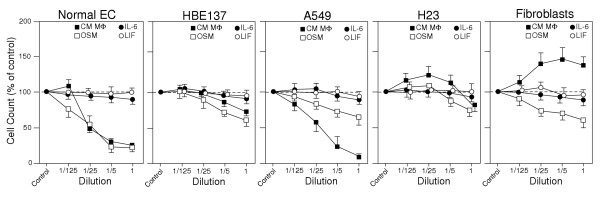
**Effect of cytokines on growth of lung cell cultures**. The cells listed at the top were cultured for 6 days in the presence of serially diluted cytokines or conditioned medium of LPS-activated macrophages (*CM MΦ*). Dilution 1 for the cytokines was 100 ng/ml and for macrophage medium a 1/10-dilution of the original conditioned medium. The number of viable cells for each culture was expressed relative to the cell number in the control culture (defined as 100%). Values represent means and S.D. of 4 separate cultures.

To relate cytokine signaling with effects on cell proliferation, representative lung cell types were cultured for 6 days in growth medium containing in addition increasing concentrations of OSM, IL-6 or LIF (Fig. 2). In all cell types, OSM reduced in a dose-dependent manner proliferation, with maximal effect at a concentration of 20 to 100 ng/ml. Maximal inhibition varied among the cell types and ranged from >80% (normal primary epithelial cells) to ~40% (HBE137, A549, fibroblasts) and ~20% (H23). IL-6 and LIF did not appreciably alter proliferation of epithelial or fibroblastic cells. The comparative analyses also indicated that immortalization of epithelial cells with the E6E7 gene (HBE4 or HBE137) or constitutive activation of ERK pathway (H23) correlated with a lower growth inhibition by OSM.

The same cells were tested for growth in the presence of conditioned medium from lipopolysaccaride (LPS) activated macrophages. Such medium is considered to contain a physiologically relevant mixture of inflammatory mediators (Table 1). The LPS macrophage medium suppressed the proliferation of epithelial cells in a dose-dependent fashion (Fig. 2). Suppression ranged from >80% (primary epithelial cells and A549) to <20% in HBE137 and H23. In contrast, the same treatment led to enhanced proliferation of fibroblasts.

**Table 1:** Cytokine production of pulmonary macrophages

Cytokine	Concentration (ng/ml)*	
	
	Minus LPS	Plus LPS
IL-1β	0.01 ± 0.01	5.4 ± 1.3
TNFα	0.4 ± 0.3	137 ± 44
IL-6	2.0 ± 0.6	290 ± 113
OSM	0.08 ± 0.01	0.87 ± 0.29
IL-8	27 ± 1	101 ± 26
IL-10	0.16 ± 0.01	8.4 ± 1.5
G-CSF	0.09 ± 0.01	20.3 ± 1.6
IL-12	0.01 ± 0.01	0.18 ± 0.03
TGFβ1	< 0.002	< 0.002

* 2 × 10^6 ^macrophages/ml incubated for 16 h with medium containing 1 μg/ml LPS. Mean and S.D., N = 3.

Taken together, these data indicated that cytokine responsiveness and growth regulation by OSM and inflammatory mediators are indeed subject to alterations in lung epithelial cells in part as a function of immortalization and transformation. Characteristic changes in the cellular response patterns may serve as markers for the transformation process or may even contribute functionally to tumorigenesis. One of the key questions is at what stage in the transformation of lung epithelial cells are these changes established. To address this question, we used short-term primary cultures of non-immortalized epithelial cells derived from normal and pathologically distinct stages of premalignant lesions sampled by brushing during bronchoscopy of patients. The experimental approach also allowed us to establish the more basic information of what is the cytokine responsiveness of normal epithelial cells and what is the range among individuals.

### Application of primary epithelial cells derived from bronchial brushing

Epithelial cell cultures were established from the brushed bronchial epithelium. Biopsies of the same sites were processed for pathological and cytological evaluation. The pathological findings (normal, metaplasia, dysplasia or non-invasive carcinoma *in situ*) were subsequently applied in the interpretation of the signaling data (Table 2). No cases of invasive carcinoma or advanced lung cancer were included. From May 2000 to March 2005, 192 separate brushings were taken from 96 patients (EC-1 to EC-96). From these, 113 brushings (59%) yielded sufficient epithelial cells that expanded to cultures suitable for functional analyses. Approximately 50% of sites initially judged to be abnormal by bronchoscopy proved to be normal by histology.

**Table 2:** Cultures of bronchial epithelial cells used in this study

	Number	Successful
Patients	96	
Brushings (total)	192	113
Paired brushings	89	43
Paired cultures		
Normal/Normal		20
Normal/Metaplasia		17
Normal/Dysplasia		2
Normal/Carcinoma		4
Single cultures		
Normal		20
Metaplasia		2
Dysplasia		1
Carcinoma		4

Based on immunostaining, every cell preparation consisted of >95% cytokeratin-positive epithelial cells. All cultures derived from normal and abnormal sites showed essentially the same epithelial cell morphology. To determine whether or not the primary cultures demonstrated any gross chromosomal abnormalities, cells were analyzed by SKY. With the exception of one carcinoma, the cells showed normal karyotypes. The carcinoma cell culture carried a chromosome 10 deletion (q11.2-q22) in all metaphase spreads.

### Paired epithelial cell cultures indicate the occurrence of altered cytokine responsiveness at early stage of transformation

**Figure 3:**
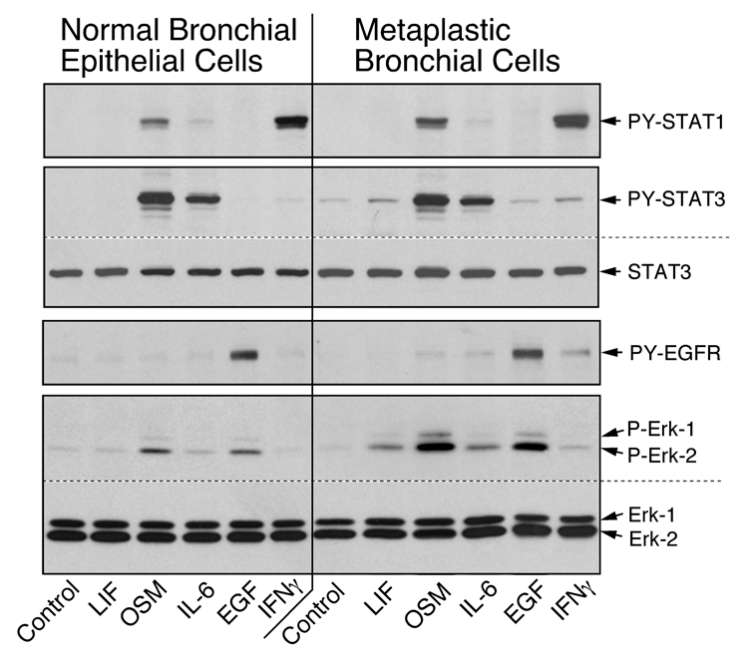
**Cytokine-specific signalling in bronchial epithelial cells**. Paired primary cultures of normal and metaplastic epithelial cells (EC-14) were treated for 15 min with the factors listed at the bottom. The relative levels of phosphorylated STAT1, STAT3, EGFR and ERK, as well as the total STAT3 and ERK, were determined by immunoblotting.

**Figure 4:**
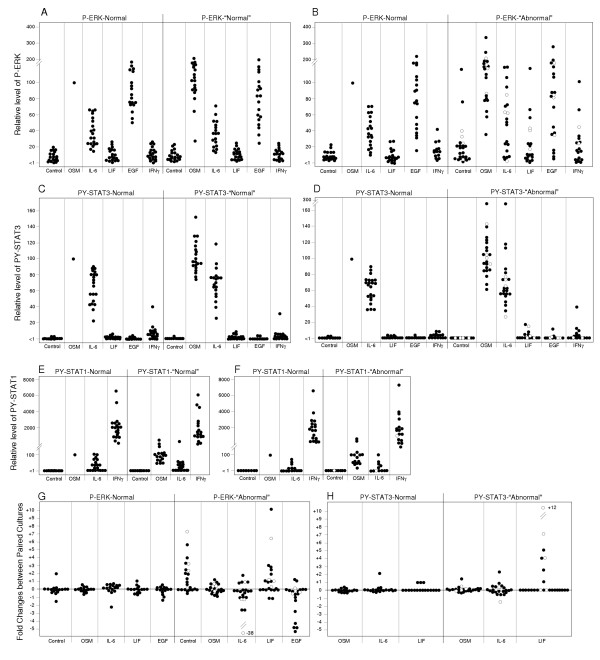
**Activation of STAT1, STAT3 and ERK signalling in paired epithelial cell cultures**. Paired cultures of epithelial cells, as listed in Table 2, were analyzed for the relative changes in STAT1, STAT2 and ERK signalling mediated by the treatments listed at the bottom. The level of phosphorylation of the signalling proteins was expressed relative to the level determined in the OSM-treated normal epithelial cells in the corresponding pair of cultures (defined as 100). **A**, **C **and **E **represent 20 pairs of the combination normal/normal and **B**, **D **and **E**, represent 23 pairs of the combination of normal/abnormal (metaplasia, dysplasia, carcinoma). **G **and **H**, indicate the fold-changes in the level of phosphorylated ERK and STAT3 in the matched of culture pairs of normal/normal (left side panel) and normal/abnormal epithelial cells. The values for the 4 carcinoma cases are shown by open circles.

The cytokine responsiveness of the epithelial cells was determined by the activation of signaling and by the effect on DNA synthesis. Signaling was measured in first passage subcultures by treatment with cytokines and growth factors for 15 min followed by immunodetection of the phosphorylated signaling proteins (representative example in Fig. 3). The level of phosphorylated STAT1, STAT3 and ERK1/2 were quantified and expressed relative to the level of these proteins in OSM-treated normal cultures in each pair (Fig. 4A–F).

The analyses of normal bronchial epithelial cells defined the following response pattern. The reaction to cytokine treatment (Fig. 3) was comparable to that of type II epithelial cells (Fig. 1A). Data from 63 separate preparations of normal bronchial epithelial cell cultures indicated that basal level of phosphorylated ERK was consistently low (7.5 ± 5.6 % of OSM level; mean ± SD) and the basal levels of phosphorylated STAT3 and STAT1 were generally low to non-detectable. OSM prominently activated ERK (Fig. 4A &4B) and STAT3 (Fig. 4C &4D). The response was uniformly high among cultures from different individuals. IL-6 response, although quite variable among individuals, was consistently below that of OSM. The level of activated ERK was 37 ± 18% of OSM (Fig. 4A &4B) and activated STAT3 was 63 ± + 27% (Fig. 4C &4D). The two-fold lower activation of ERK relative to STAT3 by IL-6 is due to the difference in signaling by the IL-6 and OSM receptor [16,22]. LIF did not elicit a measurable ERK and STAT3 signaling in any of normal epithelial cell culture. EGF activated ERK to 90 ± 29% of OSM level (Fig. 4A &4B). EGF generally had no measurable effect on STAT3 even when the cells were treated with EGF for longer than 15 minutes (not shown). In all cases, IFNγ did not appreciably activate ERK and STAT3, but yielded highest level of STAT1 phosphorylation (2441 ± 2073% of OSM level (Fig. 4E &4F). The IFNγ response from patient to patient showed the highest variability among the cytokine responses, even though paired cultures from individual patients exhibited highly consistent levels of STAT1 activation by IFNγ.

The reproducibility of detecting comparable patterns in independently derived cell cultures was assessed in those paired cultures in which the samples were initially classified as "abnormal" (or "suspicious") by autofluorescence bronchoscopy but proved normal by subsequent pathological analysis (Fig. 4A,C &4E). When cell cultures were derived from histologically normal areas with abnormal fluorescence, the level and range of signaling reactions elicited by the treatments were essentially identical to those with normal histology and normal fluorescence. ERK activation by OSM was 108 ± 35%, by IL-6 was 31 ± 15%, and by EGF was 85 ± 40%. STAT3 activation by OSM was 97 ± 31% and by IL-6 was 63 ± 27%. The cytokine-induced activation of ERK and STAT3 in paired cultures indicated highly consistent response patterns (Fig. 4G and 4H, left side panels). Confirmed premalignant epithelial cells (metaplasia, dysplasia and carcinoma) exhibited two recurring aberrant phenotypes when compared with normal cells. The first aberrancy was the elevated basal or cytokine-stimulated level of phosphorylated ERK (Fig. 4B and 4G, right panel) and the second was the reactivation of LIFR function (Fig. 4D and 4H, right panel).

In the 23 preneoplastic cell cultures, the basal level of phosphorylated ERK was increased to 22 ± 27% of the OSM value (p = 0.07). Based on two-sample Kolmogorov-Smirnov test, two cases that were cultured from metaplastic foci were above the 95% confidence interval. Preneoplastic cells showed also an overall trend of increased ERK signaling when treated with OSM (133 ± 66%; p = 0.17) or IL-6 (53 ± 38%; p = 0.01) but not with EGF (85 ± 67%; p = 0.46). The ERK signaling by IL-6 and EGF included several cases that lie outside of the 95% confidence interval. For the IL-6 response, 3/23 cases were above and 5/23 cases below the 95% confidence interval, and for EGF response, 4/23 cases were above and 3/23 cases below. STAT3 phosphorylation by OSM and IL-6, and STAT1 phosphorylation by OSM, IL-6 and IFNγ did not show significant differences among the cases tested (Fig. 4C–F), in contrast to ERK phosphorylation.

**Figure 5:**
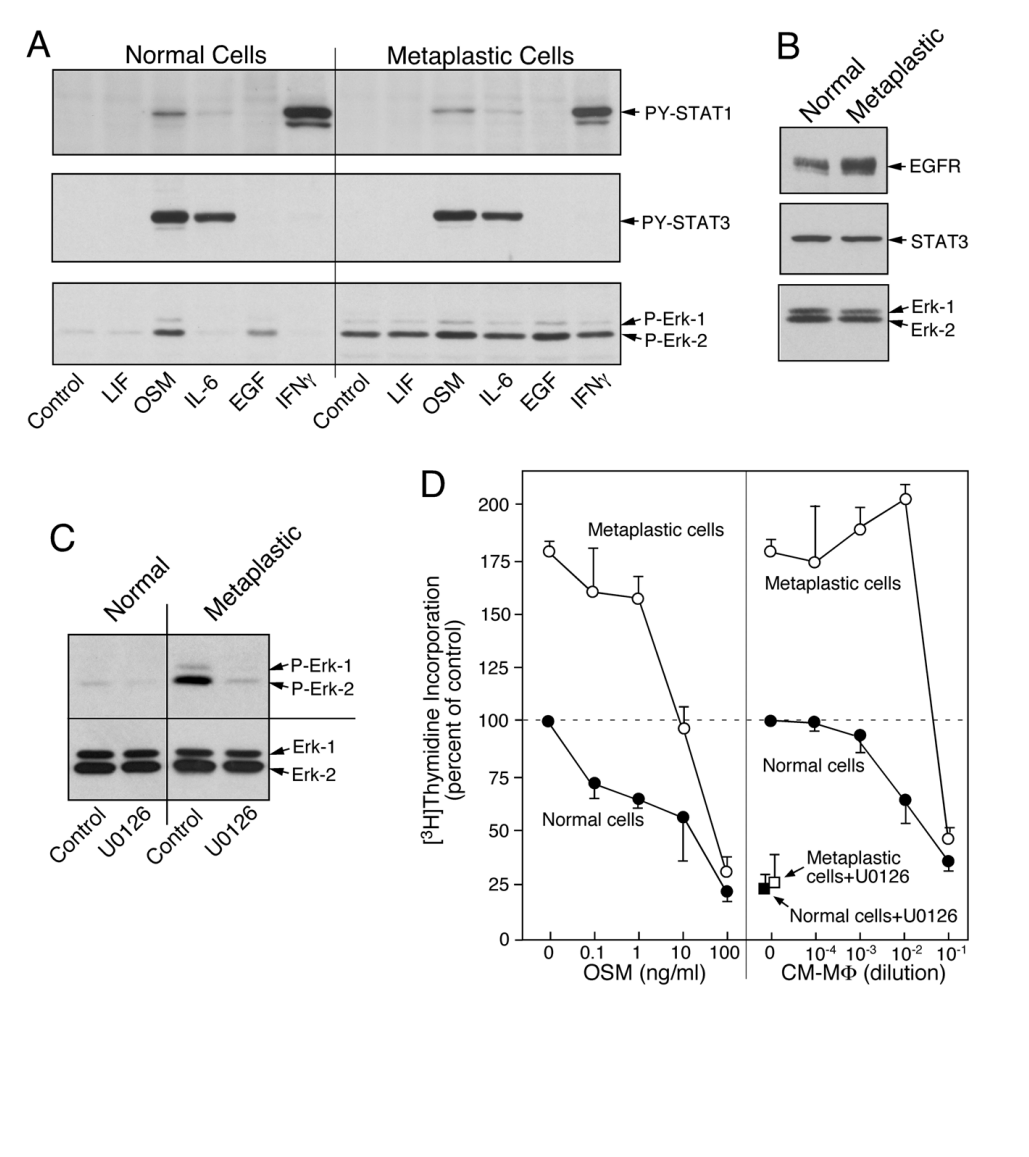
**Deregulated ERK signalling in metaplastic epithelial cells**. **A**. Paired cultures of epithelial cells (EC-85) were analyzed by immunoblotting for cytokine-mediated phosphorylation of STAT1, STAT3 and ERK. Basal level of phosphorylated ERK but not of phosphorylated STATs is elevated. **B**, Expression of EGFR, STAT3 and ERK in the untreated cultures was determined by immunoblotting. **C**, Cultures of normal and metaplastic cells were treated for 3 h with serum-free RPMI containing 0.1% carrier DMSO or the same medium with 10 μM U0126. The level of phosphorylated ERK was determined by immunoblotting. **D**, DNA synthesis was determined in response to treatment with serially diluted OSM and conditioned medium of LPS activated macrophages. One set of cultures of normal and metaplastic cells were also treated for 3 hour with 10 μM U0126 in normal growth medium prior to the addition of [^3^H]thymidine. The incorporation of [^3^H]thymidine (mean and range of duplicate cultures) were normalized to the number of cells and expressed relative to the values determined for the control cultures of the normal epithelial cells.

Among the abnormal epithelial cells with elevated basal ERK phosphorylation (Fig. 4B), the two cell cultures from metaplastic foci in the airway were distinct because the basal ERK phosphorylation that was equivalent to the OSM-treated control culture (Fig. 5A). The increased phosphorylation of ERK, but not of STATs in these metaplastic cells was similar to the regulatory phenotype of NCI H23 tumor cell line (Fig. 1A). Due to the elevated ERK phosphorylation, the response to EGF was obscured, even though a normal or even enhanced level of EGFR could be demonstrated (Fig. 5B). The high basal level phosphorylation of ERK was abrogated by treatment with U0126, an inhibitor for MEK1 (Fig. 5C), suggesting a constitutive activation of the MAPK pathway upstream of ERK in these metaplastic cells.

**Figure 6:**
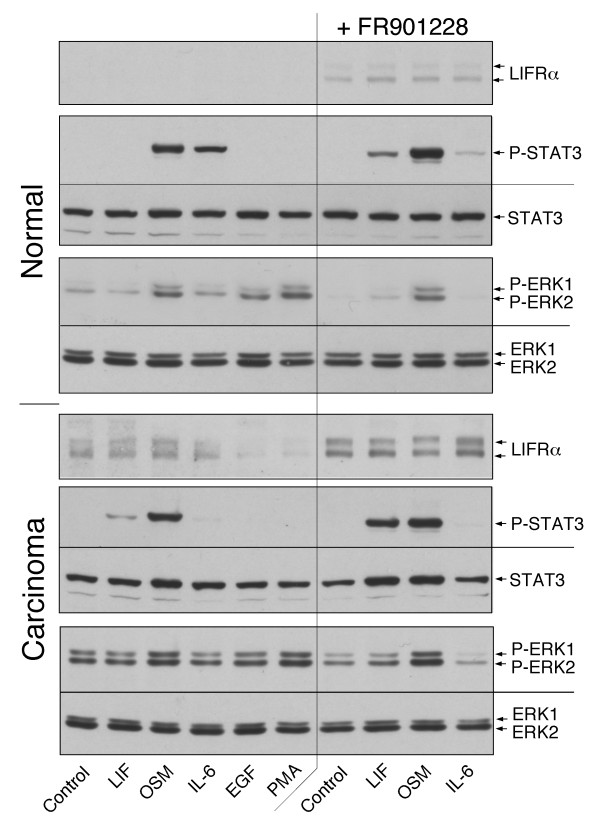
**Regulated expression of LIFR**. Paired cultures of normal and carcinoma cells (EC-9) were incubated with or without 20 nM FR901228 for 6 h and then treated for 15 min with the factors listed at the bottom. The level of phosphorylated and total STAT3 and ERK and total LIFRα were determined by immunoblotting.

The pair-wise comparison also revealed that 5/23 of the abnormal cases (4 metaplasia and 1 carcinoma) had a LIF response detectable by the activation of ERK and STAT3 (Fig. 3, Fig. 4B,D,G and 4H). Three cases (2 metaplasia and 1 carcinoma) produced a signaling that lied outside of the 95% confidence interval. The carcinoma case also included a prominent reduction of IL-6 receptor function (Fig. 6), which resulted in an overall cytokine response pattern comparable to that of the NCI H125 tumor cell line (Fig. 1A). The changes in signaling reactions correlated with receptor expression. The expression of LIFRα in normal cells was undetectable by immunoblotting whereas expression was observed in the 5 cultures of abnormal cells with LIF response.

The re-expression of LIFR in various cell types is controlled by an epigenetic process that depends on histone acetylation within CpG islands of the LIFR promoter region [22]. To determine whether altered protein acetylation could account for the switch in LIF responsiveness from normal to carcinoma cells, the normal epithelial cells were treated for 6 h with the histone deacetylase inhibitor FR901228. The treatment induced the expression of LIFRα and established a LIF-dependent STAT3 and ERK signaling that was comparable to the level observed in the carcinoma cells (Fig. 6). A similar FR901228 treatment of the carcinoma cells further enhanced LIFR expression and LIF-dependent signaling (Fig. 6). Interestingly, the same treatment reduced he expression of IL-6R in normal cells resulting in a response pattern as found in the corresponding carcinoma cells from the same patient (Fig. 6).

### OSM and secreted macrophage factors attenuate proliferation of bronchial epithelial cells

**Figure 7:**
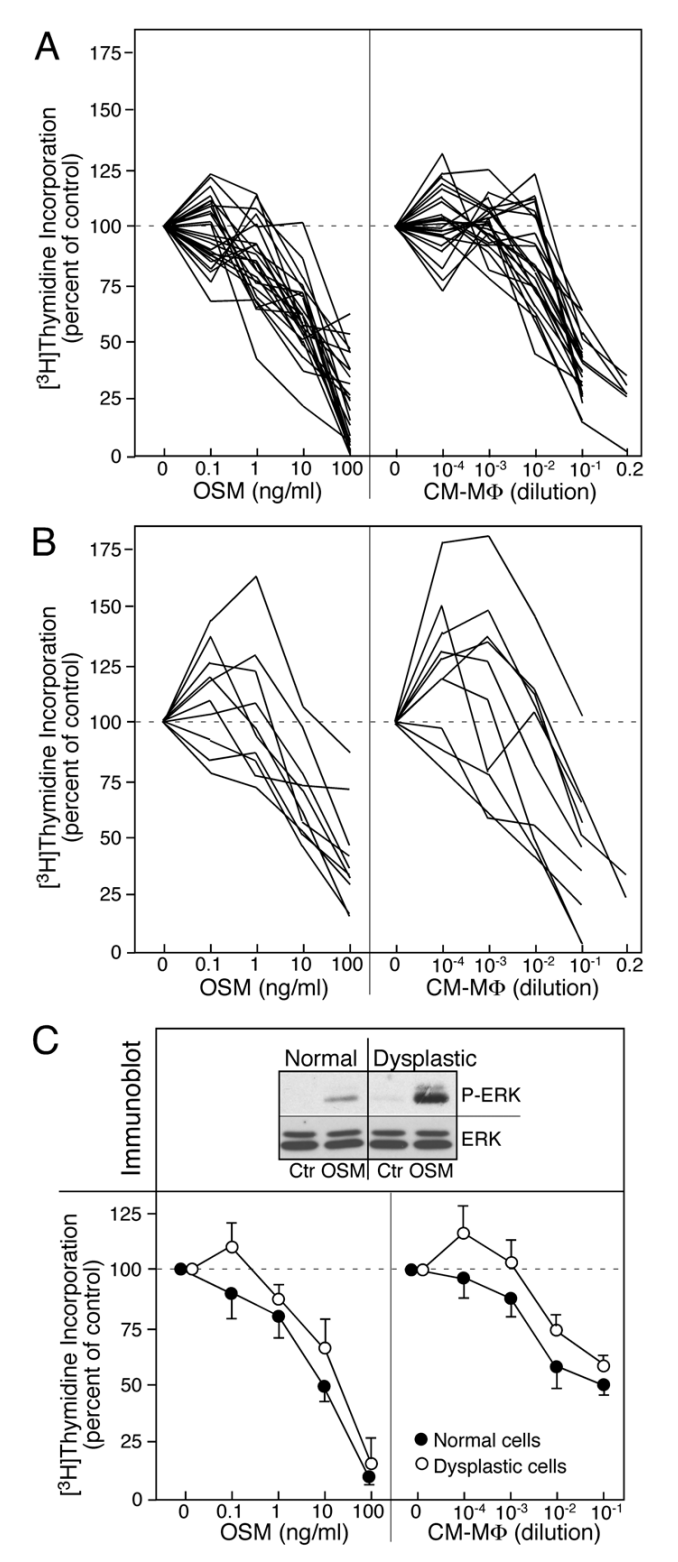
**Effect of OSM and macrophage factors on DNA synthesis of normal and abnormal epithelial cells**. A, Normal epithelial cells (25 cultures) and B abnormal epithelial cells (8 metaplasia and 2 dysplasia) were treated with serially diluted OSM or conditioned macrophage medium and the incorporation of [^3^H]thymidine determined. All values expressed relative to the untreated cultures in each series. C, Paired cultures of normal and dysplastic epithelial cells (EC-65) were analyzed by immunoblotting for OSM-stimulated ERK phosphorylation and the same cells for inhibition of [^3^H]thymidine incorporation by OSM and macrophage-factors (mean and range of duplicate values)

DNA synthesis of the epithelial cells was inhibited by OSM and LPS conditioned medium from of activated macrophages in a dose-dependent fashion (Fig. 7). The data for all cultures identified as normal cells (Fig. 7A) indicated that low concentrations of OSM (≤ 0.1 ng/ml) or LPS conditioned macrophage medium (<10^-3 ^dilution) had no appreciable modulating effects. Only at higher concentrations, suppression of DNA synthesis became evident (p < 0.05). In contrast, several cultures of metaplastic and dysplastic cells exhibited the trend, in which the cells responded to low doses of OSM or conditioned medium from macrophages by a stimulation of DNA synthesis (Fig. 7B). The exact sign test using the paired samples (normal and abnormal) indicated a significant increase of DNA synthesis by low dose (10^-3 ^dilution) of macrophage medium (p = 0.016). The comparison of the data from paired cultures by the Wilcoxon two-sample test also indicated that DNA synthesis in the abnormal epithelial cells was significantly increased at lowest concentration of OSM (p = 0.02) and macrophage medium (p = 0.005) relative to the normal cells. The reduced sensitivity for inhibited DNA synthesis in all cases could be correlated with metaplasia which shows enhanced ERK phosphorylation in response to cytokine treatment (example of dysplastic cells in Fig. 7C). In addition, we noted that in premalignant cells the basal ERK phosphorylation approached the level of OSM-treated cells or EGF-treated cells (Fig. 4B). In addition, the thymidine incorporation by premalignant cells maintained in growth medium was close to two-fold higher than in the corresponding normal cultures (Fig. 5D). As expected, the ERK activity was reduced by treatment in both normal and carcinoma cells with U0126 which correlates with the suppression of DNA synthesis observed (Fig. 5C). High concentrations of OSM or macrophage factors were effective to reduce this intrinsically stimulated DNA synthesis in both normal and premalignant epithelial cell cultures.

## Discussion

This study addressed the relationship of inflammation and oncogenic transformation of lung epithelial cells. We sought to identify cellular changes in IL-6 cytokine signaling that correlate with attenuated growth suppression of lung cancer cells. To our knowledge, this is the first report of a functional comparison of cytokine response in short-term primary epithelial cell cultures generated from brushings of normal and abnormal bronchial epithelial sites as characterized by autofluorescence bronchoscopy. We were able to demonstrate for the first time that (a) the response profile of normal epithelial cells and is variable among individuals, and that (b) premalignant epithelial cells already have stable changes in receptor activities for IL-6 cytokines and in signal transduction involving the ERK pathway. Similar changes in the signaling reactions are found in established lung cancer cell lines and these correlate in part with less inhibited cell proliferation.

The interpretation of the results requires the understanding of three underlying issues: (i) the use of primary cell cultures to define the regulatory phenotypes of cells that are representative of premalignant lesion *in situ*; (ii) the potential mechanism that alters signalling reactions; and (iii) the causal relationship of altered signaling with the pattern of gene expression and growth regulation in abnormal lung cells.

### Primary cultures of bronchial epithelial cells as representative of specific lesions

Our hypothesis was that the responsiveness of premalignant epithelial cells to inflammatory mediators and IL-6 cytokines is altered and that these changes permit sustained proliferation in the presence of inflammation. The target of our functional analyses was the proliferating premalignant cells of the central epithelium, in patients who had not yet developed lung cancer. All of the patients have a history of smoking, resulting in the presence of tar deposition and corresponding accumulation of tissue macrophages that harbor the ingested tar particles. We used short-term cultures of proliferating epithelial cells derived from foci of premalignant change as identified by autofluorescence bronchoscopy.

Our goal was to culture abnormal proliferating cells that were representative of a specific lesion identified in the airway with autofluorescence. A tissue sample collected by a focal biopsy provides lesion-specific cellular material for culture, which contains an admixture of epithelial cells, basal cells, fibroblasts and stromal cells [24]. In our collection process, the brush moves across the lesion, collecting a site-specific sample of abnormal cells devoid of non-epithelial cells. When taken as a whole, the cultured cells from the foci of premalignancy in the airway behaved differently than cultured cells from a normal area in the same patient. Our functional analyses (Figs. 3,4,5,6,7) indicated a consistent cytokine response pattern, which has been used as a marker for the phenotype of normal bronchial epithelial cells. The quantitative range of responses observed among preparations likely reflects the individual variation of epithelial cells due to genetic and cellular differentiation differences. We believe that bronchial brushings are optimal for recovering relatively homogenous cultures of proliferating epithelial cells free of macrophages and stromal cells.

As noted above, the cultures of cells obtained from the brushing of abnormal sites may include cells in different stages of transformation as well as some normal epithelial cells. We did find that the relative recovery and growth rate of viable cells from brushings of normal epithelium and from pre-malignant epithelial lesions were comparable *in vitro*. It is conceivable however that normal cells, if included in the brushing of abnormal sites, could outperform the transformed cell types. An experimental assessment of homogeneity included SKY analysis. In all instances, cells from normal, metaplastic and dysplastic sites proved to be diploid, which is consistent with the findings of Franklin et al. [24]. Since we can not rule out a fractional contribution of normal epithelial cells to the response profile determined for abnormal cells (Fig. 4B,D,F,G and 4H) the results may underestimate the altered phenotype of transformed cells.

We know that regulatory phenotypes may have been influenced by cellular changes introduced by growth *ex vivo*, and this creates an unavoidable ambiguity of the tissue culture approach. To normalize for this possibility, the comparison of normal and abnormal cells adhered to a strict side-by-side analysis of cultures, which had been generated and maintained under identical conditions. The differences discovered by this approach are considered to describe the effect of the transformation process on cytokines responsiveness (Fig. 4G and 4H) as well as proliferation (Fig. 7).

The sites for brushings representing normal and abnormal epithelium were solely selected based on the visual appearance when viewed during bronchoscopy, under two illumination conditions. For each of the 96 patients, the pathological examination of the sample taken from normal sites confirmed the normal phenotype of the epithelium. In contrast, the examination of the tissue from sites with grossly abnormal fluorescence suggested that only ~50% of the cases involved metaplasia or worse. Epithelial cell cultures from areas of abnormal fluorescence that did not exhibit preneoplasia under histological examination normal represented functionally normal epithelium, despite occasional microscopic evidence of inflammation. The patterns of cytokine signaling and proliferation observed for those cultures were fully compatible with the normal epithelial phenotype (Fig. 4G &4H). It is conceivable that the causes for the abnormal appearance of the epithelial sites involved transient processes in the bronchial environment. But the deviation of such sites from normal epithelium with an altered phenotype could not be detected *in vivo *with our analytical tools. The manifestation of these processes could be lost during the establishment of primary cell cultures.

### Mechanisms of altered responsiveness

In the tissue culture amplification of cells from brushings, the regulatory phenotype represents a stable feature. The epithelial cell responsiveness to cytokines is subject to effective positive and negative feedback mechanisms that could significantly alter the receptor-mediated signaling, depending on cytokine exposure. Reduction of signaling by IL-6 cytokines has been associated with treatment-induced receptor protein down regulation [16,25], expression of signal modifying SOCS proteins [26] or desensitization of gp130 [27]. Enhanced signalling can be the consequence of treatment-induced expression of receptor proteins [16]. All of these reversible regulatory events are presumed to be resolved during *in vitro *culture period required to establish the primary cells cultures. Treatment of the cells at this point should provide the most accurate reflection of the signaling capability of the cytokine receptor systems and the effect on gene expression and proliferation.

The difference between normal and preneoplastic cell types goes beyond the range of differences observed for duplicate samplings of normal epithelium (Fig. 4), and is ascribed to the stable modification brought about by the transformation process. Possible mechanisms are genetic and epigenetic changes [1,28] that lead to the inactivation of receptor genes such as silencing LIFR by DNA methylation [29] or enhanced expression of LIFR, OSMR, but reduced expression of IL-6R by acetylation of histone and transcription-controlling factors [22]. Whether enhanced signaling through LIFR or IL-6R is also a consequence of gene amplification as noted to be the case for EGFR [30], is not known. Thus far, no example of a duplication of the LIFR gene in transformed cells has been reported.

Aside of altered receptor expression, changes in any of the downstream signal-transducing proteins and enzymes are likely to contribute to the signaling phenotype of the lung cells. For instance, no STAT3 signaling by IL-6 cytokine receptors is found in the prostate PC3 cells because these cells have lost the genes for STAT3 and STAT5 [31]. None of the primary cultures or lung cancer cell lines revealed such a drastic modification of signaling as a function of transformation. A more common observation in lung cancer is the enhanced signaling by IL-6 cytokine receptors towards the STAT and ERK pathway (Figs. 1,3,4,5). While immunoblot analysis did not indicate appreciable changes in the expression levels of these signal-transducing proteins to account for enhanced signaling, further work is needed to investigate two other possible mechanisms. First, enhanced signaling might result from altered level of protein kinases (JAKs, MAPKs), which determine the engagement of the signaling pathways, and secondly, it is possible that phosphatase levels might determine the extent and duration of STAT and MAPK signaling process [14,32].

The constitutive activation of the ERK pathway as well as the enhanced signaling through ERK by both IL-6 cytokines and EGF is the most notable feature in about one quarter of the metaplastic and carcinoma lesions (Fig. 4B &4G). This phenotype, including its U0126 inhibition, points to an aberration of the MAPK pathway that may involve the more commonly found oncogenic mutation of Ras [33]. Alternatively, as already suggested above, a reduced dephosphorylation of ERK by a deficiency in appropriate MAPK phosphatase activities could be involved. These possibilities remain to be identified in cells derived from premalignant lesions.

### Altered cytokine receptor signaling as cause for changed cellular response

The profile of gene expression and cellular proliferation are downstream events from the altered signaling by IL-6 cytokines in premalignant epithelial cells. The deregulation of ERK and enhanced proliferation of epithelial cells has been noted in many cancer cell types [23,34], and ERK deregulation appears to also cause the reduced suppression by OSM and inflammatory cytokines (Fig. 5D &7). The mode of action may be twofold: enhancing expression of mitogenic functions (e.g., immediate growth response genes) and the reduced expression of cell cycle arresting proteins (p21 and p27) [14,35]. The precise mode of OSM mediated growth suppression is still unclear. It has been proposed that enhanced STAT3 activity is oncogenic [36]. However, a mutant gp130 that is unable to activate STAT3 also fails to suppress of proliferation, suggesting STAT3 is a potential inhibitor of growth [37]. Studies on OSM action on different breast cancer cell lines cytokines have suggested that the balance between STAT and ERK activation (magnitude and duration) determines growth promotion or growth inhibition [37].

## Conclusion

Short-term cultures of bronchial epithelial cells can be established from brushings obtained at bronchoscopy. Analyses of paired cultures of cells derived from normal and abnormal site permits the identification of the responsiveness of the cells to inflammatory mediators. Normal epithelial cells show a highly consistent and strong signaling response to OSM, IL-6, and IFNγ and EGF. Early stage of premalignant transformation is associated with modified activities of signal transduction pathways activated by cytokines. Most prominent alterations include elevated ERK phosphorylation and re-expression and function of LIF receptor. OSM and the factors released by activated pulmonary macrophages suppress proliferation of normal epithelial cells. However, this suppression is significantly reduced in abnormal cells. These cytokine responses in cultured preneoplastic cells are remarkably similar to those seen in established lung cancer cell lines. The data suggest that a change in the signaling reaction to inflammatory mediators as a function of transformation contributes to the capability of lung tumor cells to proliferate in presence of tumor-associated inflammation.

## List of abbreviations

EGF, epidermal growth factor; IL-6, interleukin-6; IFNγ, interferon γ LIF, leukemia inhibitory factor; LIFR, leukemia inhibitory factor receptor; ERK, extracellular regulated kinase; LPS, lipopolysaccharides; MAPK, mitogen-activated protein kinase; OSM, oncostatin M; PBS, phosphate buffered saline; SKY, spectral karyotyping; STAT, signal transducer and activator of transcription.

## Competing interests

The author(s) have no competing financial or non-financial interests.

## Authors' contributions

GML performed all patient-related aspects of the work; ET and FB carried out all cellular and molecular characterizations of the primary cells cultures; DT performed all pathological examinations of biopsies; SR managed the clinical and research data; JY performed all statistical analyses; SM applied SKY analysis to epithelial cell cultures; and HB established the experimental cell system. GML and HB designed the study and drafted the manuscript. All authors read and approved the final manuscript.

## Pre-publication history

The pre-publication history for this paper can be accessed here:



## References

[B1] Driscoll KE, Carter JM, Howard BW, Hassenbein DG, Pepelko W, Baggs RB, Oberdorster G (1996). Pulmonary inflammatory, chemokine, and mutagenic responses in rats after subchronic inhalation of carbon black. Toxicol Appl Pharmacol.

[B2] Ardies CM (2003). Inflammation as cause for scar cancers of the lung. Integr Cancer Ther.

[B3] Philip M, Rowley DA, Schreiber H (2004). Inflammation as a tumor promoter in cancer induction. Semin Cancer Biol.

[B4] Lam S, Kennedy T, Unger M, Miller YE, Gelmont D, Rusch V, Gipe B, Howard D, LeRiche JC, Coldman A, Gazdar AF (1998). Localization of bronchial intraepithelial neoplastic lesions by fluorescence bronchoscopy. Chest.

[B5] Saccomanno G, Archer VE, Auerbach O, Saunders RP, Brennan LM (1974). Development of carcinoma of the lung as reflected in exfoliated cells. Cancer.

[B6] Auerbach O, Saccomanno G, Kuschner M, Brown RD, Garfinkel L (1978). Histologic findings in the tracheobronchial tree of uranium miners and non-miners with lung cancer. Cancer.

[B7] Bota S, Auliac JB, Paris C, Metayer J, Sesboue R, Nouvet G, Thiberville L (2001). Follow-up of bronchial precancerous lesions and carcinoma in situ using fluorescence endoscopy. Am J Respir Crit Care Med.

[B8] Hirsch FR, Prindiville SA, Miller YE, Franklin WA, Dempsey EC, Murphy JR, Bunn PA, Kennedy TC (2001). Fluorescence versus white-light bronchoscopy for detection of preneoplastic lesions: a randomized study. J Natl Cancer Inst.

[B9] Breuer RH, Pasic A, Smit EF, van Vliet E, Vonk Noordegraaf A, Risse EJ, Postmus PE, Sutedja TG (2005). The natural course of preneoplastic lesions in bronchial epithelium. Clin Cancer Res.

[B10] Knaapen AM, Borm PJ, Albrecht C, Schins RP (2004). Inhaled particles and lung cancer. Part A: Mechanisms. Int J Cancer.

[B11] Emmendoerffer A, Hecht M, Boeker T, Mueller M, Heinrich U (2000). Role of inflammation in chemical-induced lung cancer. Toxicol Lett.

[B12] Grant SL, Begley CG (1999). The oncostatin M signalling pathway: reversing the neoplastic phenotype?. Mol Med Today.

[B13] Kortylewski M, Heinrich PC, Mackiewicz A, Schniertshauer U, Klingmuller U, Nakajima K, Hirano T, Horn F, Behrmann I (1999). Interleukin-6 and oncostatin M-induced growth inhibition of human A375 melanoma cells is STAT-dependent and involves upregulation of the cyclin-dependent kinase inhibitor p27/Kip1. Oncogene.

[B14] Heinrich PC, Behrmann I, Haan S, Hermanns HM, Muller-Newen G, Schaper F (2003). Principles of interleukin (IL)-6-type cytokine signalling and its regulation. Biochem J.

[B15] Kim H, Baumann H (1999). Dual signalling role of the protein tyrosine phosphatase SHP-2 in regulating expression of acute-phase plasma proteins by interleukin-6 cytokine receptors in hepatic cells. Mol Cell Biol.

[B16] Blanchard F, Wang Y, Kinzie E, Duplomb L, Godard A, Baumann H (2001). Oncostatin M regulates the synthesis and turnover of gp130, leukemia inhibitory factor receptor alpha, and oncostatin M receptor beta by distinct mechanisms. J Biol Chem.

[B17] Loewen G, Reid M, Tan D, Klippenstein D, Nava E, Natarajan R, Mahoney M (2004). Bimodality Lung Cancer Screening in High risk Patients. Chest.

[B18] Travis WD, Colby TV, Corrin B, Shimosato Y, Brambilla E (1999). World Health Organization classification of lung and pleural tumors.

[B19] Matsui S., Sait S, Jones CA, Nowak N, Gross KW (2002). Rapid localization of transgenes in mouse chromosomes using a combined Spectral Karyotyping/FISH technique. Mammalian Genome.

[B20] Tan D, Kirley S, Li Q, Ramnath R, Slocum H, Brooks J, Wu CL, Zukerberg L (2003). Loss of Cables Protein Expression in Human Non-Small Cell Lung Cancer: A Tissue Microarray Study. Human Pathol.

[B21] Bepler G, Koehler A, Kiefer P, Havemann K, Beisenherz K, Jaques G, Gropp C, Haeder M (1988). Characterization of the state of differentiation of six newly established human non-small-cell lung cancer cell lines. Differentiation.

[B22] Blanchard F, Kinzie E, Wang Y, Duplomb L, Godard A, Held WA, Asch BB, Baumann H (1228). FR90 an inhibitor of histone deacetylases, increases the cellular responsiveness to IL-6 type cytokines by enhancing the expression of receptor proteins. Oncogene.

[B23] Douglas WG, Tracy E, Tan D, Yu J, Hicks WL, Rigual NR, Loree TR, Wang Y, Baumann H (2004). Development of head and neck squamous cell carcinoma is associated with altered cytokine responsiveness. Mol Cancer Res.

[B24] Franklin WA, Fokvord JM, Varella-Garcia M, Kennedy T, Proudfoot S, Cook R, Dempsey EC, Helm K, Bunn PA, Miller YE (1996). Expansion of bronchial epithelial cell populations by in vitro culture of explants from dysplastic and histologically normal sites. Am J Resp Cell Mol Biol.

[B25] Blanchard F, Duplomb L, Wang Y, Robledo O, Kinzie E, Pitard V, Godard A, Jacques Y, Baumann H (2000). Stimulation of leukemia inhibitory factor receptor degradation by extracellular signal-regulated kinase. J Biol Chem.

[B26] Lang R, Pauleau AL, Parganas E, Takahashi Y, Mages J, Ihle JN, Rutschman R, Murray PJ (2003). SOCS3 regulates the plasticity of gp130 signalling. Nat Immunol.

[B27] Mahboubi K, Kirkiles-Smith NC, Karras J, Pober JS (2003). Desensitization of signalling by oncostatin M in human vascular cells involves cytoplasmic Tyr residue 759 in gp130 but is not mediated by either Src homology 2 domain-containing tyrosine phosphatase 2 or suppressor of cytokine signalling 3. J Biol Chem.

[B28] Huber RM, Stratakis DF (2004). Molecular oncology – perspectives in lung cancer. Lung Cancer.

[B29] Blanchard F, Tracy E, Smith J, Chattopadhyay S, Wang Y, Held WA, Baumann H (2003). DNA methylation controls the responsiveness of hepatoma cells to leukemia inhibitory factor. Hepatology.

[B30] Hirsch FR, Varella-Garcia M, Bunn PA, Di Maria MV, Veve R, Bremmes RM, Baron AE, Zeng C, Franklin WA (2003). Epidermal growth factor receptor in non-small-cell lung carcinomas: correlation between gene copy number and protein expression and impact on prognosis. J Clin Oncol.

[B31] Clark J, Edwards S, Feber A, Flohr P, John M, Giddings I, Crossland S, Stratton MR, Wooster R, Campbell C, Cooper CS (2003). Genome-wide screening for complete genetic loss in prostate cancer by comparative hybridization onto cDNA microarrays. Oncogene.

[B32] Morrison DK, Davis RJ (2003). Regulation of MAP kinase signalling modules by scaffold proteins in mammals. Annu Rev Cell Dev Biol.

[B33] Ramakrishna G, Sithanandam G, Cheng RY, Fornwald LW, Smith GT, Diwan BA, Anderson LM (2000). K-ras p21 expression and activity in lung and lung tumors. Exp Lung Res.

[B34] Chang F, Steelman LS, Lee JT, Shelton JG, Navolanic PM, Blalock WL, Franklin RA, McCubrey AJ (2003). Signal transduction mediated by the Ras/Raf/MEK/ERK pathway from cytokine receptors to transcription factors: potential targeting for therapeutic intervention. Leukemia.

[B35] Klausen P, Pedersen L, Jurlander J, Baumann H (2000). Oncostatin M and interleukin 6 inhibit cell cycle progression by prevention of p27kip1 degradation in HepG2 cells. Oncogene.

[B36] Yu H, Jove R (2004). The STATs of cancer--new molecular targets come of age. Nature Rev Cancer.

[B37] Lai CF, Ripperger J, Wang Y, Kim H, Hawley RB, Baumann H (1999). The STAT3-independent signalling pathway by glycoprotein 130 in hepatic cells. J Biol Chem.

[B38] Li C, Ahlborn TE, Kraemer FB, Liu J (2001). Oncostatin M-induced growth inhibition and morphological changes of MDA-MB231 breast cancer cells are abolished by blocking the MEK/ERK signalling pathway. Breast Cancer Res Treat.

